# Severe Sepsis and Septic Shock Associated with Chikungunya Virus Infection, Guadeloupe, 2014

**DOI:** 10.3201/eid2205.151449

**Published:** 2016-05

**Authors:** Amélie Rollé, Kinda Schepers, Sylvie Cassadou, Elodie Curlier, Benjamin Madeux, Cécile Hermann-Storck, Isabelle Fabre, Isabelle Lamaury, Benoit Tressières, Guillaume Thiery, Bruno Hoen

**Affiliations:** Centre Hospitalier Universitaire, Pointe-à-Pitre, France (A. Rollé, K. Schepers, E. Curlier, B. Madeux, C. Hermann-Storck, I. Fabre, I. Lamaury, G. Thiery, B. Hoen);; Cellule Interrégionale d’Epidémiologie Antilles-Guyane, Institut de Veille Sanitaire, Gourbeyre, France (S. Cassadou);; Centre d’Investigation Clinique 1424, Institut National de la Santé et de la Recherche Médicale, Pointe-à-Pitre (B. Tressières, B. Hoen);; Université des Antilles, Faculté de Médecine Hyacinthe Bastaraud, Pointe-à-Pitre (G. Thiery, B. Hoen)

**Keywords:** chikungunya, CHIKV, severe sepsis, septic shock, vector-borne infections, viruses, Guadeloupe, French West Indies

## Abstract

During a 2014 outbreak, 450 patients with confirmed chikungunya virus infection were admitted to the University Hospital of Pointe-à-Pitre, Guadeloupe. Of these, 110 were nonpregnant adults; 42 had severe disease, and of those, 25 had severe sepsis or septic shock and 12 died. Severe sepsis may be a rare complication of chikungunya virus infection.

In November 2013, the first autochthonous cases of chikungunya virus (CHIKV) infection were identified in the territory of Saint-Martin in the French West Indies (*1*). Since that time, local transmission of the virus has been identified in nearly all Caribbean islands and in Central and South America (*2*). In Guadeloupe, an outbreak started in the first weeks of 2014 and ended by November 2014. No new definite case of chikungunya has been reported since January 2015. During the 2014 outbreak, ≈40% of the population (≈160,000 persons) became infected with CHIKV. However, the hospitalization rate for chikungunya was <0.5%. A total of 450 patients with CHIKV infection and a positive reverse transcription PCR (RT-PCR) test result for CHIKV were admitted to the University Hospital of Pointe-à-Pitre (UHPAP), Guadeloupe, and were hospitalized >24 hours. Of these 450 patients, 241 were children, 99 were pregnant women, and 110 were nonpregnant adults. The objectives of our study were 1) to describe the characteristics of nonpregnant adult patients who had atypical or severe forms of the disease and 2) to search for predictive factors for severe forms.

## The Study

During the outbreak, a standardized case report form was filled out for each patient admitted to UHPAP who had clinical symptoms consistent with chikungunya and a positive CHIKV RT-PCR test result on a blood sample. The information recorded included demographics, preexisting comorbidities, which were summarized by using the Charlson index (*3*) and McCabe classification (*4*), and clinical manifestations described by organ system (e.g., cardio-circulatory, cerebral, respiratory, renal, and hepatic). Standard definitions were used for organ system failures and severe sepsis and septic shock (*5*).

The following laboratory parameters were retrieved from the patient’s record in the hospital’s electronic medical system: whole and differential leukocyte and platelet counts; corpuscular hemoglobin; and C-reactive protein, serum creatinine, alanine aminotransferase, aspartate aminotransferase, creatine kinase, and lactate dehydrogenase levels. For each parameter, 2 values were considered, the first value recorded within the first 24 hours of admission and the most abnormal value observed from hospital day 2 through day 7. After hospital discharge or death, each case of CHIKV infection was categorized as one of the following: 1) a common form, in which only fever or arthralgia occurred; 2) an atypical form, in which >1 organ system was involved; and 3) a severe form, in which the patient had >1 organ system failure or had been admitted to the intensive care unit.

Of the 110 nonpregnant adults hospitalized with chikungunya who had a positive CHIKV RT-PCR test result, 34 had a common form, 34 had an atypical form, and 42 had a severe form. Overall, the characteristics of patients with common and atypical forms were similar. Therefore, we compared the characteristics of the 48 patients with severe forms with those of the 68 patients with nonsevere forms (i.e., common and atypical forms) by selected demographic, clinical, laboratory, and outcome characteristics. Patients with severe forms were not older and did not have more comorbidities than patients with nonsevere forms. At hospital admission, the rates of classical signs of chikungunya, such as fever and peripheral arthralgia, were similar in both groups. Patients with severe forms had significantly less occurrence of headache but increased occurrence of acute cardiac failure; they also had occurrence of organ dysfunction significantly more often than did patients with nonsevere forms (Table 1). As for laboratory abnormalities, patients with severe forms had significantly higher whole leukocyte counts, polymorphonuclear cell counts, and serum lactate dehydrogenase levels at baseline and within the first week after admission (Table 1).

**Table 1:** Selected characteristics of 110 nonpregnant adult patients with chikungunya virus infection, by severity of disease, University Hospital of Pointe-à-Pitre, Guadeloupe, French West Indies, January–November 2014*

Characteristic	Nonsevere, n = 68	Severe, n = 42	p value†
Baseline characteristics			
Median age, y (interquartile range)	71 (59–80)	68 (58–77)	0.27
Male sex	36 (53)	26 (62)	0.36
Preexisting comorbid conditions			
Immune suppression	4 (6)	1 (2)	0.69
Diabetes mellitus	28 (41)	16 (38)	0.75
Chronic heart disease	9 (14)	11 (28)	0.07
Cerebrovascular disease	6 (9)	1 (3)	0.35
Chronic lung disease	1 (2)	2 (5)	0.66
Chronic liver disease	0	1 (3)	0.8
Chronic renal disease	5 (8)	4 (10)	0.95
Cancer	5 (8)	2 (5)	0.92
Charlson index, median (interquartile range)	4 (3–5)	4 (1–5)	0,41
McCabe class 1	50 (77)	24 (62)	0.09
Clinical symptoms, present on admission to hospital			
Arthralgia/arthritis	49 (82)	30 (83)	0.84
Headache	21 (39)	3 (9)	0,002
Fever	61 (92)	35 (83)	0.25
Myalgia	24 (48)	13 (41)	0.41
Cardiac manifestations	10 (15)	28 (67)	<0.001
Central nervous system manifestations	20 (30)	13 (31)	<0.001
Respiratory manifestations	9 (14)	29 (71)	<0.001
Hepatic manifestations	1 (2)	12 (29)	<0.001
Renal manifestations	12 (19)	20 (50)	0.001
Organ failures, at any time of the course of the disease			
Cardio-circulatory failure	0	22 (52)	<0.001
Neurologic failure	0	6 (14)	0.006
Respiratory failure	0	21 (50)	<0.001
Liver failure	0	9 (21)	<0.001
Renal failure	0	15 (36)	<0.001
Laboratory data, median (interquartile range)			
Whole leukocytes, day 1, G/L	5.80 (4.00–7.00)	7.50 (4.90–13.00)	0.01
Whole leukocytes, days 2–7, G/L	3.00 (2.10–5.40)	8.70 (3.20–14.70)	<0.001
Polymorphonuclear neutrophils, day 1, G/L	4.16 (2.82–5.43)	5.59 (3.40–10.94)	0.01
Polymorphonuclear neutrophils, days 2–7, G/L	1.63 (1.05–5.02)	7.13 (2.32–11.82)	<0.001
Hemoglobin, day 1, g/dL	12.6 (11.3–13.6)	12.1 (10.2–13.5)	0.25
Hemoglobin, days 2–7, g/dL	11.9 (11.3–13.6)	8.6 (10.5–11.6)	<0.001
Platelets, day 1, G/L	178(125–233)	147 (107–199)	0.13
Platelets, days 2–7, G/L	160 (110–200)	108 (75–189)	0.04
C-reactive protein, day 1, mg/L	36 (20–79)	43 (16–75)	0.57
C-reactive protein, days 2–7, mg/L	70 (33–106)	94 (37–167)	0.24
Lactate dehydrogenase, day 1, IU/L	286 (207–354)	579 (310–1135)	0.001
Lactate dehydrogenase, days 2–7, IU/L	363 (248–418)	422 (335–1600)	0.04
Creatine kinase, day 1, IU/L	268 (132–808)	395 (237–740)	0.36
Creatine kinase, day 2, IU/L	973 (163–2826)	683 (240–2164)	0.85
Aspartate aminotransferase, day 1, IU/L	34 (25–48)	50 (30–127)	0.01
Aspartate aminotransferase, days 2–7, IU/L	28 (19–58)	64 (33–351)	0.2
Alanine aminotransferase, day 1, IU/L	20 (15–30)	31 (21–57)	0.02
Alanine aminotransferase, days 2–7, IU/L	28 (19–58)	40 (20–234)	0.1
Creatinine, day 1, µmol/L	95 (78–131)	119 (81–182)	0.07
Creatinine, days 2–7, µmol/L	101 (81–139)	153 (75–410)	0.09
Outcome			
Death	1 (2)	13 (31)	<0.001

*Values are no. (%) except as indicated. All patients were hospitalized and had infection confirmed by reverse transcription PCR. †p value denotes the comparisons between the 42 patients with severe forms and the 68 patients with nonsevere forms, based on nonparametrical tests.

Among the 42 patients who had a severe form of the disease, 25 patients had illness consistent with the case definition for severe sepsis and had no other identified cause for this syndrome but CHIKV, according to blood and urine cultures, which had been performed systematically in all these patients. Overall, the background characteristics of these 25 patients were not significantly different from those of the other 85 patients (Tables 1, 2). At admission to hospital, these 25 patients had significantly higher occurrence of cardiac, respiratory, and renal manifestations and had significantly higher leukocyte counts and levels of serum lactate dehydrogenase, aspartate aminotransferase, and creatinine, which are clinical and laboratory indicators of sepsis, than did patients without severe sepsis or septic shock (Table 2). In addition, their mortality rate was significantly higher than that in patients without severe sepsis or septic shock (48% vs. 3%, p<0.001).

**Table 2:** Selected characteristics of 110 nonpregnant adult patients with chikungunya virus infection, by presence or absence of sepsis or septic shock, University Hospital of Pointe-à-Pitre, Guadeloupe, French West Indies, January–November 2014*

Characteristic	Severe sepsis or septic shock, n = 25	No severe sepsis or septic shock, n = 85	p value†
Baseline characteristics			
Median age, y (interquartile range)	70 (59–77)	70 (59–78)	0.966
Male sex	17 (68)	45 (53)	0.252
Preexisting comorbid conditions			
Immune suppression	1 (4)	4 (5)	1
Diabetes mellitus	10 (40)	34 (40)	1
Chronic heart disease	6 (26)	14 (17)	0.374
Cerebrovascular disease	1 (4)	6 (8)	1
Chronic lung disease	2 (9)	1 (1)	0.124
Chronic liver disease	1 (4)	0 (0)	0.223
Chronic renal disease	3 (13)	6 (8)	0.414
Cancer	1 (4)	6 (7)	1
Charlson index, median (interquartile range)	4 (3–5)	4 (2–5)	0.579
McCabe class 1	9 (39)	21 (26)	0.296
Clinical symptoms, present on admission to hospital			
Arthralgia/arthritis	19 (91)	60 (80)	0.347
Headache	3 (17)	21 (30)	0.376
Fever	21 (84)	75 (90)	0.467
Myalgia	8 (44)	29 (45)	1
Cardiac manifestations	20 (80)	18 (22)	<0.001
Central nervous system manifestations	8 (32)	25 (30)	1
Respiratory manifestations	18 (72)	20 (24)	<0.001
Hepatic manifestations	7 (28)	6 (8)	0.012
Renal manifestations	17 (71)	15 (19)	<0.001
Organ failures, at any time of the course of the disease			
Cardio-circulatory failure	17 (68)	5 (6)	<0.001
Neurologic failure	3 (12)	3 (4)	0.129
Respiratory failure	14 (56)	7 (8)	<0.001
Liver failure	6 (24)	3 (4)	0.004
Renal failure	13 (52)	2 (2)	<0.001
Laboratory data, median (interquartile range)			
Whole leukocytes, day 1, G/L	8.10 (6.10–13.10)	5.80 (4.10–7.10)	0.004
Whole leukocytes, days 2–7, G/L	10.70 (3.40–15.00)	3.05 (2.35–7.00)	<0.001
Polymorphonuclear neutrophils, day 1, G/L	5.96 (4.48–11.02)	4.30 (2.82–5.91)	0.01
Polymorphonuclear neutrophils, days 2–7, G/L	8.73 (2.55–12.02)	1.80 (1.09–5.86)	<0.001
Hemoglobin, day 1, g/dL	12.1 (10.6–13.5)	12.5 (11.2–13.5)	0.796
Hemoglobin, days 2–7, g/dL	10.6 (7.9–11.7)	11.7 (10.3–12.9)	0.013
Platelets, day 1, G/L	139 (106–192)	176 (123–233)	0.063
Platelets, days 2–7, G/L	104 (59–189)	149 (108–200)	0.03
C-reactive protein, day 1, mg/L	46 (20–75)	40 (20–79)	0.856
C-reactive protein, days 2–7, mg/L	92 (41–204)	70 (33–106)	0.119
Lactate dehydrogenase, day 1, IU/L	606 (288–946)	310 (226–401)	0.007
Lactate dehydrogenase, days 2–7, IU/L	422 (346–1600)	363 (266–422)	0.05
Creatine kinase, day 1, IU/L	653 (304–1394)	264 (140–639)	0.08
Creatine kinase, day 2, IU/L	911 (357–3932)	727 (163–2642)	0.283
Aspartate aminotransferase, day 1, IU/L	60 (39–127)	33 (24–48)	0.001
Aspartate aminotransferase, days 2–7, IU/L	125 (36–626)	54 (35–104)	0.044
Alanine aminotransferase, day 1, IU/L	33 (24–57)	20 (15–31)	0.02
Alanine aminotransferase, days 2–7, IU/L	56 (28–499)	30 (19–61)	0.055
Creatinine, day 1, µmol/L	157 (114–343)	96 (77–134)	0.008
Creatinine, days 2–7, µmol/L	226 (123–570)	101 (76–141)	0.002
Outcome			
Death	12 (48)	2 (3)	<0.001

*Values are no. (%) except as indicated. All patients were hospitalized and had infection confirmed by reverse transcription PCR. †p value denotes the comparisons between the 25 patients with severe sepsis or septic shock and the 85 patients with no severe sepsis or septic shock, based on nonparametrical tests.

The following case report describes one of the 25 patients with severe sepsis or septic shock. The patient died of septic shock, which had no other identified cause but CHIKV infection.

An 85-year-old man with no prior medical history except treated hypertension developed an acute influenza-like syndrome. On day 2 of illness, a common form of CHIKV infection was diagnosed by his general practitioner, and the patient received treatment for his symptoms. On day 4, he was referred to a hospital emergency department because of persistent high-grade fever. His hemodynamic condition was normal, and the diagnosis of CHIKV infection was maintained; however, because of elevated levels of whole leukocytes (total 40 G/L), polymorphonuclear neutrophils (37.5 G/L), and serum C-reactive protein (170 mg/L), blood and urine samples were collected for culture, and treatment with ceftriaxone was started. Approximately 12 hours after admission to the emergency department, the patient experienced onset of septic shock and died within 4 hours. All blood and urine cultures were negative for CHIV. A PCR test for leptospirosis also was negative. A CHIKV-positive RT-PCR test result was the only positive diagnostic test result obtained for this patient.

## Conclusions

Although chikungunya usually has a mild course, severe life-threatening complications can develop during the acute phase of the disease (*6*,*7*). Previous studies indicate that the disease can be complicated by severe multiple organ failure and lead to death (*8*,*9*). Very recently, the first cases of severe sepsis and septic shock that could be attributed to CHIKV infection were reported (*10*,*11*). In some of these cases, acral skin necrosis was observed (*11*).

The replication of viruses, especially of the family *Herpetoviridae*, has been shown to occur frequently during the course of septic shock syndromes of bacterial origin, not only as a stress-induced reactivation but also as a superinfection causing additional morbidity (*12*). By contrast, cases of virus-triggered septic shock have been reported only rarely (*13*), although a recent cross-sectional study of septic shock syndromes in a pediatric population suggested that viruses might be the only etiology in up to 10% of cases (*14*). On the other hand, genuine acute severe viral infections might be complicated with a bacterial septic shock, which is well known to occur in cases of influenza but has also been reported in cases of arboviral diseases, such as dengue fever (*15*).

In our study, none of the 25 patients who had a positive CHIKV RT-PCR test result and a severe sepsis or septic shock syndrome early in the course of chikungunya had another organism identified as a potential cause of sepsis. This finding strongly suggests that CHIKV can, in rare cases, cause severe sepsis and septic shock syndromes, an observation that had not been reported until very recently. Additional studies are needed to identify any background characteristics that might be associated with the onset of severe sepsis or septic shock.
